# Metal and metalloid concentrations in wild mammals from SW Europe: European hedgehog (*Erinaceus europaeus*) and badger (*Meles meles*)

**DOI:** 10.1007/s11356-023-30615-4

**Published:** 2023-11-03

**Authors:** Javier García-Muñoz, Nunzio Antonio Cacciola, Federico Plazzi, María Prado Míguez-Santiyán, Francisco Soler Rodríguez, Ana López-Beceiro, Luis Eusebio Fidalgo, Salomé Martínez-Morcillo, Marcos Pérez-López

**Affiliations:** 1 Toxicology Area, Faculty of Veterinary Medicine (Universidad de Extremadura), 10003 Cáceres, Spain; 2 Department of Veterinary Medicine and Animal Production, University of Naples Federico II, Via F. Delpino 1, 80137 Naples, Italy; 3 Department of Biologia Evoluzionistica Sperimentale, University of Bologna, Via Selmi 3, 40126 Bologna, Italy; 4 Department of Veterinary Clinical Sciences, Faculty of Veterinary Medicine (USC), 27003 Lugo, Spain

**Keywords:** Biomonitoring, Potentially toxic elements, European hedgehog, European badger, Liver, Kidney

## Abstract

In recent years, there have been increasing ecological and global concerns associated to Potentially Toxic Elements (PTEs). Thus, the relevance of wild mammals as biomonitors has been globally recognised. In the present study, Cd, Pb, Hg, Zn and As concentrations were quantified in European hedgehog and badger inhabiting SW Europe, and cumulative trends in relation to age and sex were evaluated. Liver and kidney samples were collected, mineralised and PTE content was determined by ICP-MS. Zn was the most abundant element quantified in both organs (239 and 89.8 mg kg^-1^ for hedgehogs and 179 and 164 mg kg^-1^ dw for badgers). In hedgehogs, very high Hg concentration were quantified (4.35 and 15.5 mg kg^-1^ dw in liver and kidney), and Cd was the most abundant for badgers (4.70 and 7.61 mg kg^-1^ dw in liver and kidney). Positive correlations were observed for the concentrations of PTE in the organs of both species. Age-dependence increased only Cd concentration, with levels in adult kidneys being significantly higher. In this study, European hedgehog and badger were used as biomonitors for the determination of PTEs to provide current reference values in relatively non-polluted areas of SW Europe, and to enhance the use of these species for future ecotoxicological studies.

**Graphical Abstract:**

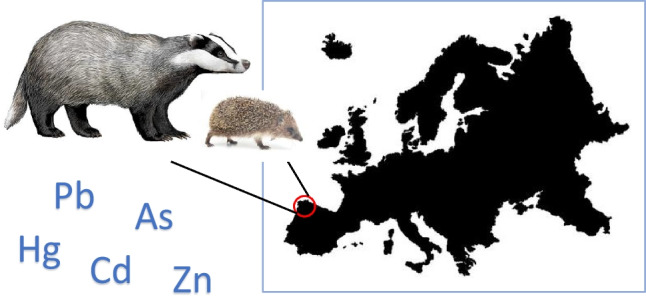

**Supplementary Information:**

The online version contains supplementary material available at 10.1007/s11356-023-30615-4.

## Introduction

Among the environmental pollutants released into ecosystems, Potentially Toxic Elements (PTEs), otherwise known as heavy metals, are highly relevant and a matter of environmental and public health concern in the last decades. According to Ali and Khan (2018), “heavy metals are naturally occurring metals having atomic number greater than 20 and an elemental density greater than 5 g cm^-3^”. Despite their natural and ubiquitous presence in the environment, they frequently occur at high concentrations as a result of human activities such as mining, farming, or industrial processing (Walker et al. 2012; Gall et al. 2015; Raj and Maiti 2020). Among PTEs, some of these metals, such as zinc (Zn), are essential elements which can mediate biological functions (i.e., metabolic reactions, the synthesis of structural proteins, hormones, or enzymes), while other metals, such as cadmium (Cd), lead (Pb), mercury (Hg), or the metalloid arsenic (As), as non-essential elements, they are not involved in any organic functions (NRC 2005; Kalisińska 2019; Ali and Khan 2019). When time of exposure and concentrations are exceeded, they constitute a potential health risk and a serious threat for the ecosystem and humans (Boening 2000; Kosik-Bogacka and Łanocha-Arendarczyk 2019; Tomza-Marciniak et al. 2019; Charkiewicz and Backstrand 2020; Osuna-Martínez et al. 2021). In fact, the risk presented by these elements is mainly a result of sub-lethal effects (carcinogenicity, nephrotoxicity, mutagenicity, teratogenicity and neurotoxicity, as well as endocrine disruption), and the substance priority list recently published by the Agency for Toxic Substances and Disease Registry (ATSDR 2022) has ranked As, Pb and Hg as the top three most hazardous substances, closely followed below by Cd. From an ecotoxicological perspective, the prevalence of these contaminants throughout the food chain, from lower to upper trophic levels, has been extensively studied (Hernández-Moreno et al. 2013; Dibbern et al. 2021; Yan et al. 2021; Zhang et al. 2021; Kalisińska et al. 2023). This has even led to the processed of bioaccumulation and biomagnification in wildlife (Chormare and Kumar 2022). Nevertheless, the accumulation and transfer of these pollutants are dependent on species, metal(loid), and toxicokinetic. In a large amount of biomonitoring studies, the determination and quantification of these elements has primarily been carried out in soft tissues such as liver and kidney. These organs are of great relevance as they are involved in detoxification mechanisms and can therefore be used to obtain insights into the effects of short and long-term metal exposure (Kalisińska 2019; Jota Baptista et al. 2022a). However, bioaccumulation trends depend on many factors, for example biological factors such as age, size, and sex, or external factors, including the degree of pollution of the site, diet, seasonal variation, or environmental factors (soil, pH, and redox conditions) (Talmage and Walton 1991; Peakall and Burger 2003; Burger 2007; Brand et al. 2020). It therefore makes it difficult to interpret the results. When considering biological factors, multiple studies have observed a positive correlation between age and bioaccumulation rate, finding PTEs such as Cd or Pb in increasing concentrations with age in wild mammals (Komarnicki 2000; Sánchez-Chardi and Nadal 2007; Pérez-López et al. 2016). However, the influence of sex on the accumulation of these elements abovementioned, due to a lack of understanding, is not clear yet as a result of the differences in hormonal and reproductive state, gene expression and metabolic rate between males and females (Burger 2007). In small and medium mammal, some studies have shown higher PTEs concentration in males than in females (Beernaert et al. 2007; Zarrintab and Mirzaei 2017), vice versa (Komarnicki 2000; Scheirs et al. 2006; Millán et al. 2008; Fritsch et al. 2010; Jota Baptista et al. 2023) or no differences (González et al. 2008; Rautio et al. 2010; Pérez-López et al. 2016).

Owing to their wide distribution, and food preferences, wild mammals are largely exposed to these chemical pollutants. Plenty of studies have shown their important role as bioindicators when considering the assessment of pollutants, including PTEs (Kalisińska 2019). More specifically, small and medium-sized mammals have an elevated metabolic rate, making them highly sensitive to metal(loid)s accumulation. They are often regarded as representative prey items for top predators making their exposure to pollutants a major pathway for the entry of these chemicals into food chains. Thus, they have been considered as potential biomonitors of environmental contamination (Talmage and Walton 1991). When considering terrestrial ecosystems, this approach is common worldwide, and the use of mammals has proven to be suitable for assessing environmental and biological predictors of PTEs exposure. For example, erinaceans such as the European hedgehog (*Erinaceus europaeus*), or the mustelid European badger (*Meles meles*), with a global distribution and key position in several trophic levels, have been used in ecotoxicological studies related to Cd, As, Pb, Hg, or Zn (Hernández et al. 1985; Van Den Brink and Ma 1998; D'Havé et al. 2005, 2006; Alleva et al. 2006; Millán et al. 2008; Rautio et al. 2010; Bilandžić et al. 2012; Ozimec et al. 2015, 2017; Heimstad et al. 2019; Kalisińska et al. 2021; Mullineaux et al. 2021; Jota Baptista et al. 2023).

Some previous studies have suggested the relevance of both species as possible bioindicators due to their high exposure to PTEs. Hedgehogs are insectivores that prey on invertebrates (earthworms, slugs, earwigs, beetles, among others) (Shore 1995; D'Havé et al. 2006). It can occupy several types of habitats, such as natural, rural and urban spaces, presents a limited home range, long life span, and its migration rated is reduced (Jota Baptista et al. 2021). Besides, badgers are mesocarnivores whose diet consists of 50-70% small rodents including hedgehogs, the rest feeding on invertebrates, grasses, or reptiles. Its habitat preference consists of a mixture of agricultural landscape, deciduous forest, or hedgerows. Moreover, both rely heavily on the soil for their grooming and burrowing habits (Jota Baptista et al. 2021; Mullineaux et al. 2021). Increased bioaccessibility can therefore be expected to affect PTE accumulation in hedgehog and badger, and the higher risk of exposure to those make them important candidates as biomonitors.

Both species are evaluated by the International Union for Conservation of Nature (IUCN) at the level of Least Concern (LC). Nonetheless, the badger is included in Annex III (Protected fauna species) of the “Convention on the conservation of European wildlife and natural habitats”, Bern Convention (ETS 104 1979). In the Iberian Peninsula, they are found over a large extend and may constitute the prey for certain species, several of which are threatened or endangered such as Iberian wolf (*Canis lupus signatus*), lynx (*Lynx pardinus*), or brown bear (*Ursus arctos arctos*). The assessment of environmental contamination by PTEs through adequate monitoring programmes is therefore essential to preserve the value and biodiversity of Mediterranean ecosystems and to assess the need for possible corrective measures (Jota Baptista et al. 2022b).

For our knowledge, only three biomonitoring studies on these two species have been reported in this region, focussing exclusively on the Protected National Park of Doñana and Portugal. These studies provide metal(loid)s accumulation only in the hepatic tissue, leaving a gap in their status in other target organs such as kidney (Hernández et al. 1985; Millán et al. 2008; Jota Baptista et al. 2022b, 2023). Thus, little is known about the status of these two species in the rest of the Iberian Peninsula, and the extent to which they are affected by PTEs contamination. It should be highlight that no current references for Hg values in these two terrestrial mammals are available. The purposes of this study were therefore (i) to determine the current exposure of European hedgehog and European badger to PTEs, specifically Hg, Cd, Pb, As and Zn, from areas of NW Spain in order to fill the gap between current and nephritic values; (ii) to assess age- and sex-specific bioaccumulation trends in the concentrations of these elements; and (iii) to establish the current pollution degree cause by these elements in the terrestrial ecosystems of the Iberian Peninsula.

## Material and methods

### Sample collection

**Fig. 1:**
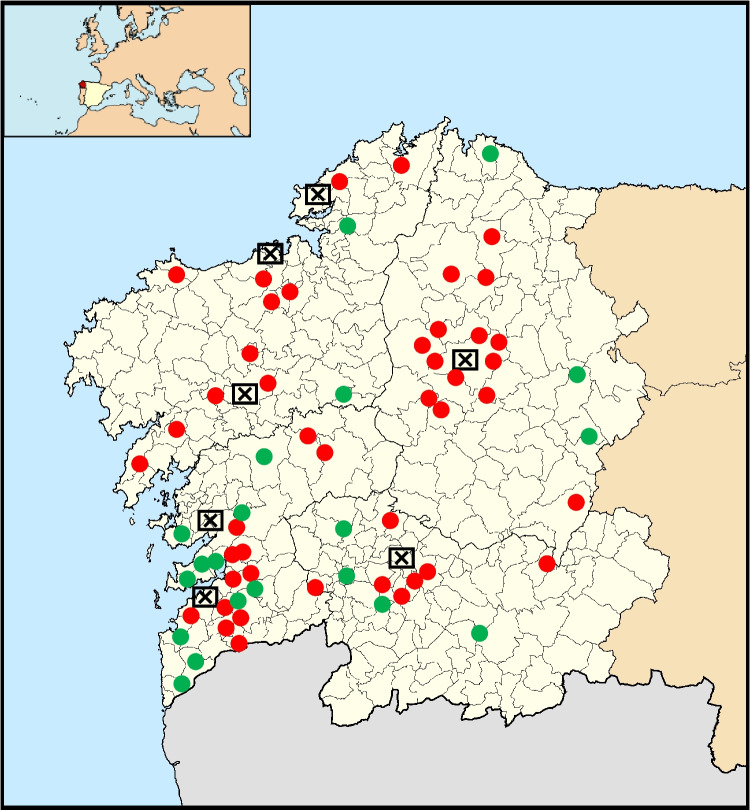
Distribution of the sampling areas for European hedgehog (  ) and badger (  ) in Galicia, northwestern Spain.  represents the main human settlement

European hedgehogs (*n* = 43; body mass -BM- = 660-1210 g) and European badgers (*n* = 20*;* BM = 4218-11000 g) were collected during the period 2020–2022 in Galicia (NW Spain) (Fig. 1). All specimens came from different locations, mainly from agricultural and forestry areas with sparse human population. The collected specimens were found recently dead or had died naturally (i.e., via infectious diseases), or because of road traffic accidents. In all cases, these animals were referred to the Wildlife Recovery Centres in this region, and only animals that had been kept at the centre for less than 5 days before dying were considered for this study. The diet provided during this period was supposed to be free of environmental contaminants. All dead specimens were frozen and stored at -80°C until sample preparation for chemical analysis, which took place at the Veterinary Faculty. The animals were sexed (hedgehog: 24 males, 16 females, 3; badger: 9 males, 11 females), and the age of the animal was estimated by the general size, the animal’s dental development and the degree of sexual maturity (hedgehog: 31 adults, 9 young; badger: 4 young, 16 adults), as previously established for different wildlife species (Pérez-López et al. 2016). Regarding hedgehogs, the sex and age of 3 individual couldn’t be determined. From each animal, internal organ samples were taken, placed in individual plastic bags and stored at -80°C. Sample preparation was carried out taking care to avoid metal contamination and losses: plastic scalpels and surgical tools were cleaned or substituted for each animal, and disposable nitrile gloves were used. The working surface was also cleaned after each operation. Samples were handled in a way to avoid any contact with metallic external surfaces (Hernández-Moreno et al. 2013).

### Metal determination

About 3-4 g of liver and kidney samples were dried in an oven for 72 h at 65° C. The PTEs levels were analysed at the Elemental and Molecular Laboratory of the Research Support Service (SAIUEX, accredited by ISO 9001:2008; University of Extremadura). 0.7 g of liver and kidney was taken for the quantification of PTEs. Sample digestion was carried out in closed Teflon PTFE flaks, following the protocol previously optimised in the laboratory for wildlife samples and using a microwave automatic digester (Nardiello et al. 2019). The concentrations of PTEs (Pb, Cd, Hg, As, Zn) were determined using an ICP-MS (7900) equipped with an autosampler (Agilent Technologies, Santa Clara, CA, USA). For all metals, the limit of detection (LOD) was 0.003 mg kg^-1^, and the limit of quantifications (LOQ) was 0.005 mg kg^-1^. All samples were run in batches that included blank and initial calibration standards. A certified sample of lyophilised bovine liver was used as reference material for quality control (BCR®, ref 185R, Community Bureau of Reference, EU). The recovery yields ranged from 84% for Cd to 96% for Zn, and the coefficients of variation were always below 10%. Final concentrations were expressed in milligrams per kilograms (mg kg^-1^) in dry weight (dw), since dry values are considered to be more reliable and consistent compared with wet weight values (Adrian and Stevens 1979). To compare PTEs concentrations measured in this study with earlier published data, other author’s results reported on wet weigh (ww) were recalculated using an average dw value of 32% for liver and 25% for kidney, respectively (Pérez-López et al. 2016).

### Statistical analysis

All data were analysed using GraphPad Prism 9.0.2 (GraphPad Software Inc., La Jolla, CA, USA). Final concentrations were expressed as mean ± standard error of mean (SEM), standard deviation (SD), median and range. Data normality was assessed by the Shapiro-Wilk test. As the data were not normally distributed, the non-parametric Kruskal-Wallis test was used to analyse the accumulation of inorganic elements in both organs. The Mann-Whitney *U* test was applied to analyse the influence of sex and age. To assess the different correlations among metal levels and tissues, Spearman’s test was used. Interpretation of the correlation strength was made based on Kalisińska et al. (2023), with the following strengths: 0.8–1 very strong; 0.6–0.79 strong; 0.4–0.59 moderate, 0.2–0.39 weak, 0–0.19 very weak. The significance level was established as *p*<0.05.

Part of statistical analyses was performed using R (R Core Team 2023). The Principal Component Analysis (PCA) was carried out using the R package FactoMineR (Lê et al. 2008) and plotted using ggplot2 (Wickham 2016) and ggbiplot (https://github.com/vqv/ggbiplot). Rows (observations) with missing entries were dropped; remaining values were log-transformed and the PCA was centred. Finally, the correlations were investigated using the corrplot (Wei and Simko 2021) R package.

## Results and discussion

In the present biomonitoring study, the concentrations of some PTEs (Pb, Cd, Hg, As, Zn) in European hedgehogs (*Erinaceus europaeus*) and European badgers (*Meles meles*) from NW Spain were assessed. The main statistical parameters related to metal concentrations (in terms of dw) in liver and kidney from both species are shown in Table 1. Based on this information, the studied elements could be detected in all samples.

**Table 1:** Main descriptive statistics corresponding to Zn, Hg, Cd, Pb and As concentrations in European hedgehog and European badger. Data are expressed in milligrams per kilograms (mg kg^-1^) dry weigh (dw)

		Element	Mean ± SEM	SD	Median	Range
European hedgehog (*n* = 43)	Liver (*n* = 43)	Zn	239 ± 17.07	112	210	18.1–441
		Hg	4.35 ± 0.706	4.63	3.00	0.19–20.4
		Cd	1.53 ± 0.334	2.19	0.68	0.01–11.0
		Pb	1.66 ± 0.478	3.14	0.62	0.15–16.4
		As	0.19 ± 0.044	0.29	0.11	0.01–1.91
	Kidney (*n* = 42)	Zn	89.8 ± 3.242	21.0	85.3	57.4–138
		Hg	15.5 ± 3.116	20.2	7.60	0.22–80.6
		Cd	5.89 ± 1.137	7.37	2.92	0.02–37.8
		Pb	0.67 ± 0.122	0.79	0.38	0.08–3.84
		As	0.20 ± 0.042	0.27	0.12	0.01–1.33
Eurasian badger (*n* = 20)	Liver	Zn	179 ± 26.50	119	142	62.2–537
		Hg	0.77 ± 0.159	0.71	0.78	0.06–3.48
		Cd	4.70 ± 1.573	7.02	2.37	0.02–25.5
		Pb	0.31 ± 0.538	0.24	0.23	0.07–1.02
		As	0.14 ± 0.079	0.35	0.06	0.02–1.64
	Kidney	Zn	164 ± 37.40	167	126	18.0–846
		Hg	1.16 ± 0.279	1.25	0.59	0.05–4.75
		Cd	7.61 ± 2.076	9.28	2.15	0.06–26.4
		Pb	0.45 ± 0.138	0.62	0.27	0.04–2.66
		As	0.08 ± 0.019	0.08	0.06	0.02–0.43

*SEM* standard error of mean, *SD* standard deviation

### Liver and kidney global concentrations

Generally, PTEs concentrations were higher in hedgehog than in badger samples, with the exception of Cd. Overall, Zn was the most abundant element in the two species in both hepatic and renal tissues (mean: 239 and 89.8 mg kg^-1^ dw, respectively, for hedgehogs; 179 and 164 mg kg^-1^ dw, for badger), being mainly stored in the hepatic tissue only for hedgehogs (*p*<0.0001). Although Zn is excreted through external tissues such as hair or spines (Vermeulen et al. 2009; Hernández-Moreno et al. 2013), the relevance of soft tissues in detoxification and storage processes makes the study of such internal tissues highly relevant. Zn is an essential element and takes part in a wide variety of physiological functions, but above the threshold values of 465 and 274 mg kg^-1^ dw for liver and kidney, respectively, in mammals, Zn becomes toxic, with several negative effects on the organism (Eisler 1993). The mean values determined in this study were lower for liver and kidney than the threshold values. Nevertheless, in badger samples, maximum Zn concentrations exceeded those values for liver (537.2 mg kg^-1^ dw) and even three times for kidney (845.7 mg kg^-1^ dw). Kosik-Bogacka and Łanocha-Arendarczyk (2019) reported the optimal Zn level in the liver and kidney of mustelid mink ranging between 83-333, and 90-100 mg kg^-1^ dw. We do not therefore dismiss the possibility that high Zn concentrations could be related to the death of these two individuals, but it may also be due to a physiological origin, such as stress, or even a response to other pollutants.

When data obtained in the present study are compared with the scientific literature, it can be concluded that Zn concentrations in hedgehog organs from NW Spain are in the range previously reported in liver (52.14 to 371 mg kg^-1^ dw) and kidney (103.2 to 135 mg kg^-1^ dw), respectively (Table 2). Our results were similar to those obtained in a hedgehog population living in unpolluted area from Finland (228.9 and 103.2 mg kg^-1^ dw) (Rautio et al. 2010). When comparing our results with previous studies performed on insectivorous small mammals in our study area, Zn levels in liver were slightly higher than those reported in the liver of wood mice (*Apodemus sylvaticus*) (158-216 mg kg^-1^ dw), Spanish shrew (*Sorex granarius*) (109 mg kg^-1^ dw), and greater white-toothed shrews (*Crocidura russula*) (229 mg kg^-1^ dw), while kidney levels were lower than the range quantified in these three small mammals (96.9 to 168, 144 and 205 mg kg^-1^ dw) (González et al. 2008). A similar reasoning could be made when comparing with the results of studies carried out with different species from the Iberian Peninsula, such as greater white-toothed shrews (167.26-232.26 mg kg^-1^ dw) (Sánchez-Chardi and Nadal 2007; Sánchez-Chardi et al. 2007). The increase of Zn in liver can be attributed to physiological regulations such as protection and/or detoxification, and elevated concentrations in mammals may be a consequence of disrupted metabolism or high metal intakes. Regarding badger samples, our own results exceeded the range for liver (<144.3 mg kg^-1^ dw) and kidney (<156.89 mg kg^-1^ dw) reported in earlier studies (Table 2). In addition, liver and kidney concentrations were greater than those measured in other terrestrial wild mammals living in the same study area, such as Iberian wolf (*Canis lupus signatus*) (77.9 and 25.81 mg kg^-1^ dw) (Hernández-Moreno et al. 2013), red fox (*Vulpes vulpes*) (77 and 17 mg kg^-1^ dw) (Pérez-López et al. 2016) and wild boar (*Sus scrofa*) (56.9 mg kg^-1^ dw for liver) (Neila et al. 2017). Although pollution episodes have not been reported in the sampling area, high levels of this metal in both species may not just be caused by bioaccumulation from plant and prey intake, but can also be due to several natural factors such as mineralogical soil composition, pH, or redox condition, or anthropogenic factor (local point source). In recent years, 57% of Zn emissions from point sources in Galicia come from thermal power plants, and small industrial developments (Giráldez et al. 2022).

**Table 2:** Comparative study of Potentially Toxic Element (PTE) concentrations in liver and kidney of European hedgehogs and European badgers in Europe

Sp.	Site	*n*		Liver					Kidney					Ref.
				Zn	Hg	Cd	Pb	As	Zn	Hg	Cd	Pb	As	
European hedgehog	Galicia, Spain	43	dw	238.6	4.35	1.53	1.66	0.194	89.8	15.53	5.89	0.673	0.204	Present study
	Doñana, Spain	5	ww	52.14	0.41	0.54	1.46							Hernández et al. (1985)
	Urbino-Pesaro, Italy	*n*<5	ww		0.06	0.34	0.79							Alleva et al. (2006)
	Flanders, Belgium	*n*>43	dw	371		13.39	10.9	0.69	135		45.17	5.2	0.58	D'Havé et al. (2006)
	Joensuu, Finland	*n*>58	dw	228.97		1.81	1.03	0.45	103.22		5.74	0.95	0.47	Rautio et al. (2010)
	Portugal	45	dw			1.57		0.13						Jota Baptista et al. (2023)
Eurasian badger	Galicia, Spain	20	dw	178.3	0.767	4.70	0.308	0.138	164.0	1.16	7.61	0.448	0.081	Present study
	The Netherlands	83	dw						96.75-157		4.90-203	1.41-3.72		Van Den Brink and Ma (1998)
	Urbino-Pesaro, Italy	*n*<5	ww		0.18	0.67	0.40							Alleva et al. (2006)
	Doñana, Spain	1	dw	144.3	0.090	0.017	0.460	nd						Millán et al. (2008)
	Croatia	3	ww		0.037	0.537	0.106	0.033		0.084	3.05	0.156	0.012	Bilandžić et al. (2012)
	Czech Republic	1	ww	36.20	0.49	1.09	2.98	0.07	28.30	0.67	2.06	1.69	0.02	Bukovjan et al. (2014)
	Croatia	29	ww			0.395	0.197				3.046	0.190		Ozimec et al. (2015)
	Croatia	29			0.140					0.183				Ozimec et al. (2017)
	Norway	8	ww	31.63	0.047	1.19	0.243	0.010						Heimstad et al. (2019)
	Poland	5	dw		0.404					0.948				Kalisinska et al. (2021)
	Northern Ireland	56	dw	113.4			1.21	1.2	83.08			1.47	1.2	Mullineaux et al. (2021)

*nd* no detected

Data are expressed in mg kg^-1^

Regarding non-essential metals, and when considering mean values, Hg was the main element accumulated in European hedgehogs in both organs (4.35 and 15.5 mg kg^-1^ dw for liver and kidney samples, respectively), being mostly stored in renal tissue (*p*<0.0001). However, when considering the badger tissues (0.77 and 1.16 mg kg^-1^ dw for liver and kidney), no significant differences were observed between organs (*p*>0.05). It must be noted that Hg, as a potentially toxic element, is released into the natural environment via industrial and agricultural activities, being highly present in many ecosystems (Boening 2000). In this research, we measured total Hg, but the most toxic and bioavailable form of this element is methylmercury (MeHg), which under the right conditions is methylated and therefore able to biomagnifies its concentration with increasing trophic position (Peterson et al. 2020).

The mean Hg levels detected in hedgehogs were above the established threshold value for serious health effects (1.1 mg kg^-1^) in liver and kidney, but below the range associated with poisoning and death in wild mammals (25-30 mg kg^-1^ for both organs) (Shore et al. 2011). Nevertheless, 6 hedgehogs were above the toxic threshold by far. The lowest observed adverse effect level (LOAEL) established for terrestrial mammals (18.1 mg kg^-1^ in liver tissue from mink in an experimental setting) reports clinical signs and death, and the hepatic Hg concentration in two hedgehogs exceeded it (Wobeser et al., 1976). These isolated cases of excessive Hg concentrations in these organs of hedgehogs could be attributed to their individual characteristics, such as problems in metal metabolism and excretion, or exposure to extreme conditions of metal pollution. When compared with similar studies, Hg concentrations detected in this study were markedly higher than those measured in hedgehog liver samples in Italy (<0.2 mg kg^-1^ dw) (Alleva et al. 2006) or in liver and kidney of Brandt hedgehogs (*Paraechinus hypomelas*) from Iran (0.066 and 0.15 mg kg^-1^ dw) (Dahmardeh Behrooz et al. 2022). To our knowledge, no information is available about the levels of Hg in terrestrial wild mammals from our reference area, as most studies have focused on Galician estuaries and coast (De La Peña-Lastra et al. 2019). However, and taking in consideration that hedgehogs are often linked to urban landscapes, sharing the same environment as pets such as dogs, comparative studies with this terrestrial species are adequate. Likewise, Hg concentrations measured in hedgehogs were markedly higher than the results reported by López-Alonso et al. (2007) in dog liver and kidney from this region, which in all cases were below 0.25 mg kg^-1^ dw. According to Eisler (2004), it seems that size is related to resistance to Hg pollution, and elevated Hg levels in hedgehogs could be associated to dietary and behavioural habits increasing the degree of exposure to Hg. But the possibility that these concentrations have been influenced by a local point or diffuse source of pollution should not be excluded, and further analyse of these results including spatial trends must be performed. In 2019, the Hg levels were above natural background levels, with industrial activity accounting for 49% of Hg emission in Galicia (Giráldez et al. 2022).

The average Hg concentrations in the liver and kidney of badgers were 3 to 8 times higher than those obtained for the same species from different areas of Europe (ranging between 0.09-0.55 mg kg^-1^ dw) (Alleva et al. 2006; Millán et al. 2008; Bilandžić et al. 2012; Ozimec et al. 2017; Heimstad et al. 2019; Kalisińska et al. 2021). Only highlights the study by Bukovjan et al. (2014), where Hg levels quantified in this species from the Czech Republic were twice as high as in the present study (aprox. 1.53 and 2.68 mg kg^-1^ dw in liver and kidney). In short, in some individuals, the concentration which cause serious health effects was exceeded, however, mean concentrations determined in our study are in accordance with the normal values established for mustelids in liver and kidney (1.0 and 0.2-0.7 mg kg^-1^ ww) (Kalisińska et al. 2021). Findings of elevated mercury (Hg) concentrations in the liver and kidneys of European hedgehogs in the NW Iberian Peninsula raise important concerns. They may indicate possible contamination of water and soil in the region. Identification of sources and assessment of water and soil quality are critical steps to address this concern.

For the current study, a significant difference was observed in Cd levels between kidney and hepatic tissues in both hedgehog and badger. Specifically higher Cd concentrations were detected in the kidney tissues, with values of 5.89 mg kg^-1^ dw for hedgehogs and 7.61 mg kg^-1^ dw for badgers. The biological half-life of Cd lies in the range of about 25-30 years, and it primarily accumulates in the kidney, an organ often first affected by its toxicity (Cooke 2011). Over time, when renal thresholds are surpassed, Cd can also accumulate in the liver (Goretti et al. 2018). Notably, detrimental effects on mammals have been reported at Cd concentrations exceeding 105 mg kg^-1^ dw in renal tissue (Tomza-Marciniak et al. 2019). Still, the concentrations found in our study remain below this toxic threshold. A recent study developed by Jota Baptista et al. (2023) suggested that in the liver of small mammals with a predominant insectivorous diet, the average Cd concentration ranged from 0.2 to 1.5 mg kg^-1^ dw. Our study found elevated levels, possibly indicating hedgehogs varied diet which might expose them to more potential sources of Cd (Jota Baptista et al. 2021). In comparison, badgers from NW Spain presented higher Cd concentration than those recorded in previous studies (from 0.017 to 3.5 mg kg^-1^ dw), but were consistent with kidney values reported in other region like Netherlands (Table 2). This discrepancy might highlight the influence of local anthropogenic activities such as mining, farming, and industrial processes on Cd levels in our study area. Interestingly, when our findings are contrasted with other wildlife studies from the same region badgers showed higher Cd concentrations than other omnivorous species such as wild boar, red fox and European wolf (Hernández-Moreno et al. 2013; Pérez-López et al. 2016; Neila et al. 2017). Therefore, the reason why the levels determined in badgers were higher than in hedgehogs may be due to food bioavailability which relies on seasonal variation. In the Mediterranean region, earthworms can be an important part of badger diets, particularly in areas with moist soils, such as northern Spain, which has a prominent Atlantic climate with consistent wetness. However, the presence of a high concentration of Cd in the environment makes earthworms a significant source of input into the local food web, as they readily bioaccumulate this element (Al Sayegh Petkovšek et al. 2014).

The mean Pb levels for kidney samples from hedgehogs and badgers were slightly similar (0.6731 and 0.4477 mg kg^-1^ dw), whereas in the liver were markedly higher in hedgehogs than in badgers (1.660 and 0.3084 mg kg^-1^ dw), indicating that Pb is significantly accumulated in the liver (*p*<0.001). Earthworms are one of the primary components of the hedgehog diet, which bioaccumulate significantly high levels of Pb and represent a potential pathway for Pb perpetuation in the trophic webs (Beyer et al. 2018). The kidney is a significant target organ and suitable bioindicator of Pb exposure, and the concentrations in soft tissues of mammals generally decrease from the kidney to the liver (Ma 2011), which has been observed for the badger samples in this study. However, it should not be ignored that 90% of the total body burden of Pb is found in bone (Talmage and Walton 1991). In all cases, the risk of exposure to wild mammals to Pb present in soils depends on the bioavailability of Pb to plants and invertebrate soil organisms used as food.

Thus, Pb is highly present in the environment due to its widespread and long-term use by humans, with lead smelters and hunting being the major sources. In general, mean concentrations did not reach the threshold which shows clinical signs of poisoning (25–35 mg kg^-1^ dw), nor that resulting in toxicosis in mammals (15 and 5 mg kg^-1^ dw in kidney and liver), but just in a couple of individuals it was clearly exceeded (13.44 and 16.44 mg kg^-1^ dw in liver) (Ma 1996). Moreover, these mean concentrations are in the normal range established for mammals in liver and kidney (<3-4 mg kg^-1^ ww) (Baranowska-Bosiacka et al. 2019). On the other hand, our results showed that 6 specimens of hedgehogs have hepatic concentration higher than the LOAELs levels in small mammals, defined in 2.7 and 5.9 mg kg^-1^ dw for liver and kidney metal burden, respectively (Shore and Douben 1994). Pb concentrations measured in the selected tissues of the European hedgehog were in the ranges quantified in other areas of Europe (aprox. 2.47 to 10.9 mg kg^-1^ dw in liver, 0.95 to 5.2 mg kg^-1^ dw in kidney), and similar results were obtained in badgers (<10 mg kg^-1^ dw in liver, 15 mg kg^-1^ dw in kidney) (Table 2). When focusing on the Mediterranean region, especially in the natural park of Doñana, these results were slightly greater than hedgehogs from this area (1.46 mg kg^-1^ dw in liver), but similar for badger (0.46 mg kg^-1^ dw) (Hernández et al. 1985; Millán et al. 2008). We assume a hypothetical reason that in this protected park, where hunting is forbidden, hedgehogs are not as exposed to ingestion of Pb through hunting bullets as in our area. In addition, given that the corpses come from road accidents, it is also possible that the concentration has been partly influenced by the high-density traffic of combustion vehicles. Furthermore, our results were higher than other polluted areas measured in small mammals with insectivore diets, such as shrews, living surrounding of an old lead/zinc mine in southern Portugal (1.17 mg kg^-1^ dw in the liver) (Marques et al. 2007). In contrast, our results are not in accordance with Sánchez-Chardi et al. (2007) who quantified 1.93 and 5.37 mg kg^-1^ dw in the liver and kidney of shrews from free-pollutant area in NE Spain. When comparing with other mesocarnivores, Pb concentrations in badger where lower than those of the red fox from NW Spain (0.81 mg kg^-1^ dw in the liver), or Egyptian mongoose and common genet from Southern Spain (1.753 and 0.652 mg kg^-1^ dw in the liver) (Millán et al. 2008; Pérez-López et al. 2016). Pb quantification in bones could have been relevant to assess whether the presence of this metal in these two species is due to temporary or permanent exposure, however, limitations in collecting samples did not allow us to perform it.

Of the non-essential PTEs, As was the least quantified and no statically significant differences between the tissues were observed, suggesting that this metalloid is equally accumulated at low levels in the liver and kidney of European hedgehog (0.1941 and 0.2040 mg kg^-1^ dw, *p*>0.05) and European badger (0.138 and 0.081 mg kg^-1^ dw, *p*>0.05). Despite the kidney being the major organ for As elimination from the body, previous studies have shown its preference to bioaccumulate in the liver (Binkowski 2019), although the underlying mechanism is still unclear. Moreover, the As accumulation pattern in mammals varies among species, organs, and locations, rendering all biomonitoring studies with this PTE of high relevance. In this study, with no exception, the levels of As found for both tissue types and both species were below 3 mg kg^-1^, the limit considered as background and with no toxicological effect on living organisms (Pereira et al. 2006). Furthermore, low levels of As were quantified in comparison to previous studies carried out in European hedgehogs. For instance, the observed values were four and three times lower than those found for hedgehog populations living in polluted areas in Flanders, Belgium (0.69 and 0.58 mg kg^-1^ dw in liver and kidney), and two times lower than those for hedgehogs from non-polluted areas in Finland (0.45 and 0.47 mg kg^-1^ dw in liver and kidney) (D'Havé et al. 2006; Rautio et al. 2010). Nevertheless, it should be noted that our results are slightly greater than those obtained in the liver of hedgehogs from Portugal (0.13 mg kg^-1^ dw). In addition, when compared with other small mammals such as wild rats (*Rattus rattus* L.) and Algerian mice (*Mus spretus*) living close to abandoned mine areas in the Iberian Peninsula, our results are also low (Pereira et al. 2006). Considering that the sampling area is unpolluted, low As concentrations are therefore to be expected. For the European badger, the results were within the range of those observed in previous studies (<1.2 mg kg^-1^ dw in liver and kidney) (Table 2). In Spain, there has been limited research on this particular PTE in terrestrial wildlife. Notably, a study conducted by Millán et al. (2008) in badgers from the Doñana National Park did not detect this element. We propose that this discrepancy can be attributed to the use of a less precise method compared to the one used in our current research, however, differences in environmental factors should not be excluded either. As far as we know, no data about the levels of As in wild terrestrial mammals from our study area are available. Few studies have been mainly carried out in livestock and domestic animals, and our results were higher than those obtained by López-Alonso et al. (2007) in dogs from urban and rural areas of Spain (aprox. 0.039 and 0.06 mg kg^-1^ dw). We hypothesised that the main reason for this difference may be that some of the badgers come from farming areas, where As has been widely used as a pesticide and fertiliser. The concentration of As in badgers samples could be related to behavioural habits, as they spend a large part of their time in their burrows, and may even be exposed to As-polluted soils as a consequence of spreading pesticides in adjacent sites.

Based on the results obtained and the average concentration of the elements quantified, these data can provide valuable information for wildlife management in the region. Elevated concentrations of Zn and Hg in these animals suggest the current problem of the health status of local ecosystems in NW Spain due to possible contamination. This could alert wildlife authorities and environmental managers to the need to investigate and address specific sources of contamination that could affect not only wildlife, but also human health, water and soil quality. Thus, these results indicate a significant concern in terms of environmental quality in northwest Spain. Zn and Hg are PTEs that can have detrimental effects on aquatic and terrestrial ecosystems. The accumulation of these metals in wild mammal tissues indicates a possible entry of contaminants into the local food chain. This could have implications for biodiversity, as chronic exposure to metals can affect the health and reproduction of local species. In addition, these data can be an early warning signal to identify specific geographic areas that may require more stringent environmental management. Finally, in terms of environmental risk assessment, the detection of elevated Hg concentrations in hedgehogs raises questions about exposure of other organisms in the region. This could lead to further investigations into the sources and routes of mercury exposure, as well as more detailed risk assessments to evaluate potential health and environmental impacts.

### Correlation study

**Fig. 2:**
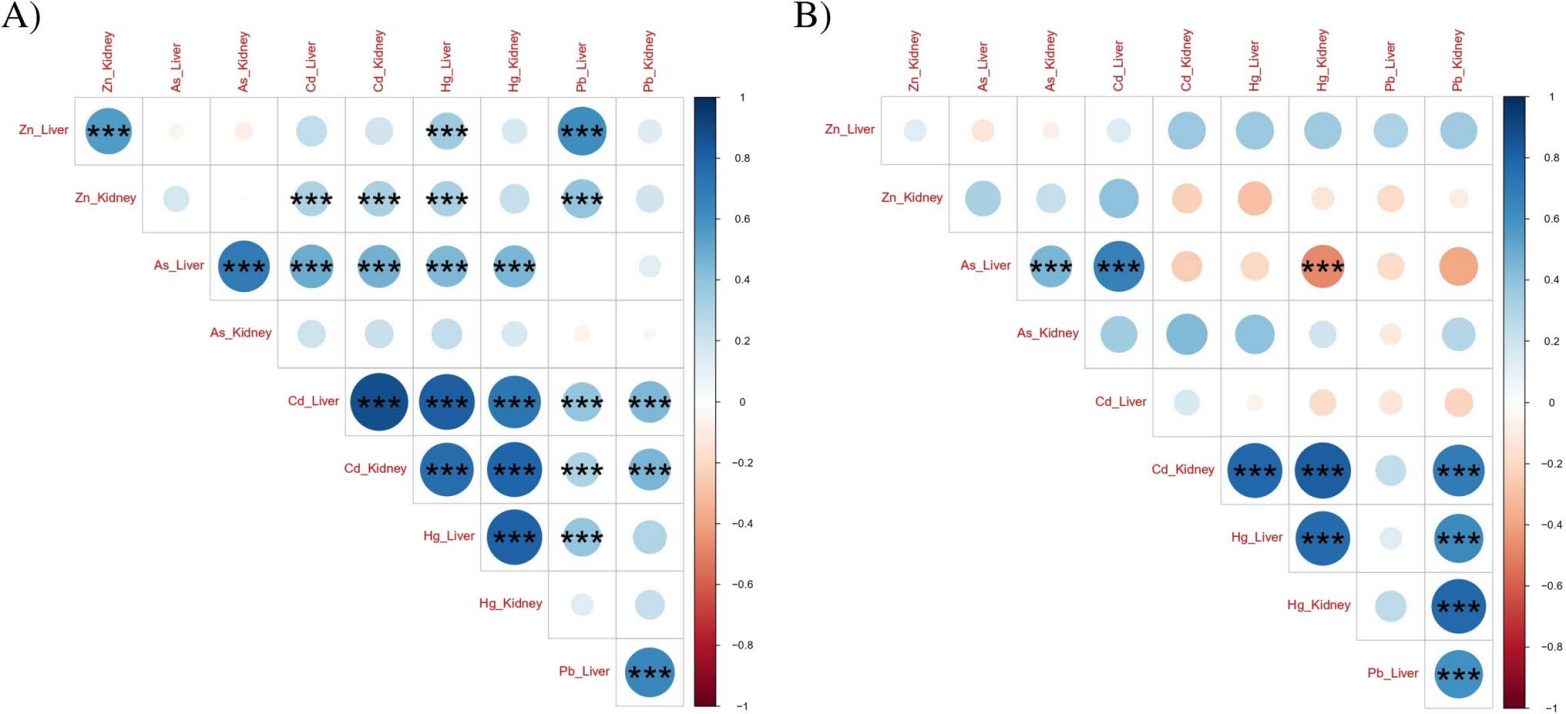
Correlation matrix of the variables analysed in the liver and kidney of European hedgehog (**A**) and European badger (**B**). Blank square means no statistically significant correlation. Blue circles are positive correlation. Red circles are negative correlation. The size of the circle marks the correlation coefficient. ***(*p*<0.001)

We observed a ‘very strong’ and ‘strong’ correlation between Hg stored in the liver and kidney of both species (*r*=0.8050, *p*<0.0001 for hedgehogs; *r*=0.7699, *p*<0.0001 for badger). Moreover, similar strong correlation between these organs was also observed for Pb in hedgehogs and badgers (*r*=0.6463, *p*<0.0001; *r*=0.6030, *p*<0.005) (Fig. 2). However, only in hedgehog, a positive very strong correlation between the levels of both organs was found for Cd (*r*=0.8786, *p*<0.0001), strong for As (*r*=0.6963, *p*<0.0001) and moderate for Zn (*r*=0.5594, *p*<0.0005). This association could be attributed to the high reabsorption rate and blood transfer, as well as similar detoxification dynamics between the liver and kidney (Boening 2000; Cooke 2011; Ma 2011). These correlations have been observed in previous studies carried out in hedgehogs and badgers, and even other wild mammals (Petrović et al. 2014; Kalisińska et al. 2021; Dahmardeh Behrooz et al. 2022).

The interaction among the metals for each tissue was also assessed. A significant very strong correlation between Cd and Hg was observed in hedgehogs for liver (*r*=0.8023, *p*<0.0001) and strong correlation in kidney samples (*r*=0.7932, *p*<0.0001) (Fig. 2). In liver samples, a positive moderate correlation between As and Cd for hedgehogs (*r*=0.5104, *p*<0.01) and strong for badgers (*r*=0.6434, *p*<0.05) was observed, as well as for As and Hg for badgers (*r*=0.6014, *p*<0.05). These results suggest that the liver (which plays a role in metal detoxification) is related to these correlations. The correlation between Cd and Hg has not been shown in hedgehogs before. However, this association is in agreement with that reported in previous biomonitoring studies. For example, these results were reported in white-toothed shrew or red fox from Spain (Sánchez-Chardi et al. 2007; Millán et al. 2008) and in river otter (*Lutra lutra*) from Hungary (Lanszki et al. 2009). In our study, a positive moderate correlation was also observed between hepatic Zn and Pb in hedgehogs (*r*=0.5973, *p*<0.0001). According to a previous study, the bioaccumulation and toxicity of heavy metals depend on the interaction between essential and nonessential metals (Neila et al. 2017). Similar to our results, positive correlations have been found between hepatic Zn and Pb levels in mammals (Millán et al. 2008; Lanszki et al. 2009). These correlations, however, seem to differ between species and tissues, making it essential to obtain a better understanding of the relationship among trace elements, their toxicokinetics and metabolism.

**Fig. 3:**
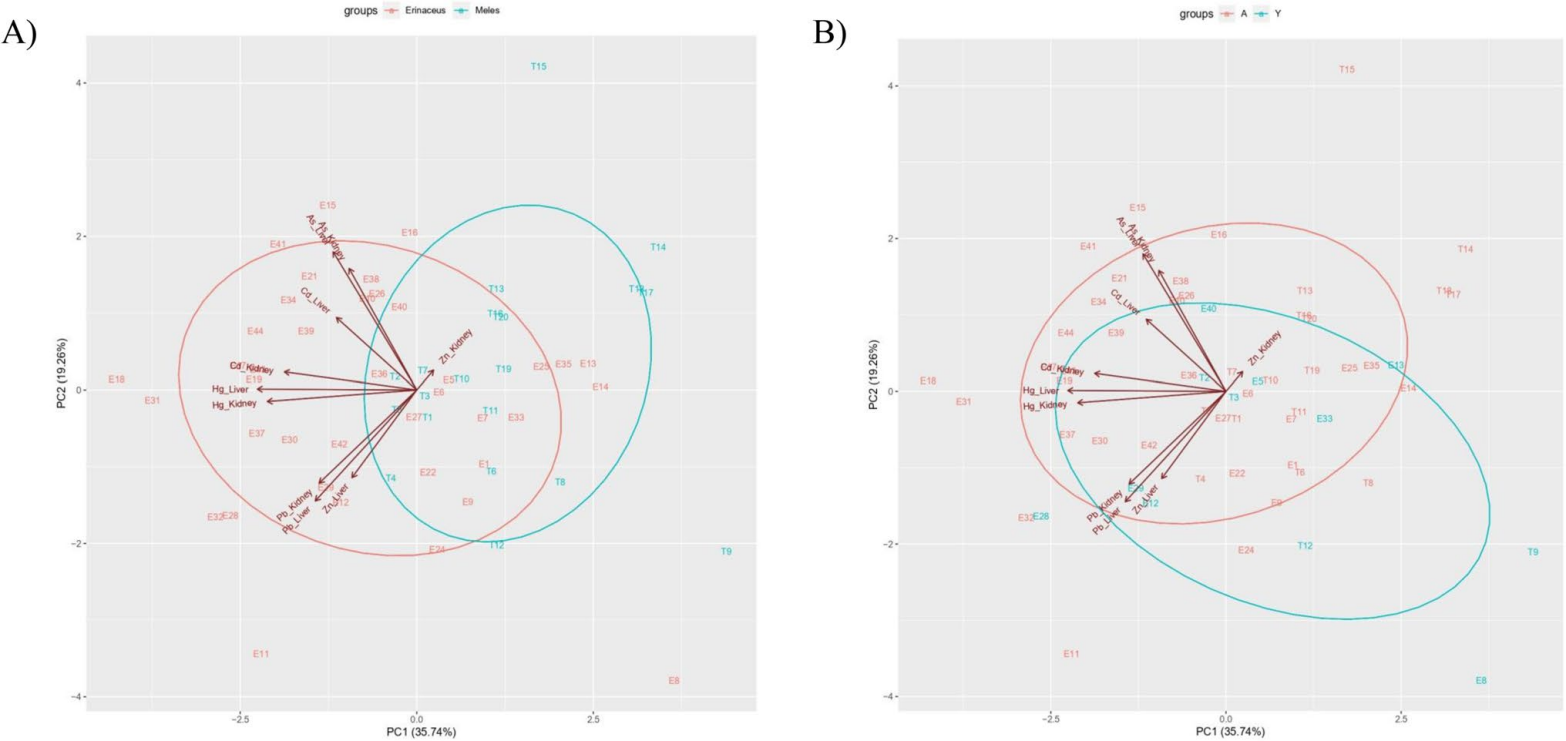
Biplot of PC1 versus PC2 with loadings of Zn, Hg, Cd, Pb and As measured in liver and kidney of 39 samples of European hedgehog and 20 samples of European Badger. The colours represent the (**A**) species (red: hedgehogs, blue: badgers), and (**B**) age (red: adults, blue: young). The total variance explained by the first two principal axes is 55%

Principal Component Analyse (PCA) was used to explore the existence of behaviour patterns in the samples and the possible correlations between variables, considering the biological factors assessed. Fig. 3 shows the plot of scores and loading of PCA obtained from the PTEs concentrations of the samples, being specified by species (Fig. 3A) and age (Fig. 3B). PCA provided the explanation of 55% of the data variance accumulated. Two principal components were extracted, the first component (PC1), which explains 35.74% of total variation, stands the strong correlation between the levels of Hg in liver and kidney. The PC1 charge was negative for all metals, mainly Hg and Cd which were the variable with the highest negative loading. For other metals, a similar trend has been observed, with the exception of Zn in the kidney, which has a positive loading. This trend in the accumulation of Hg, as abovementioned, is due to the fact that both organs are involved in Hg detoxification, and from a biological point of view, both display a similar distribution pattern (Kalisińska 2019). The second principal component (PC2), which explains 19.26% of the variation, reflects the correlation of As and Pb in both organs, being the variables with the highest negative loadings for Pb and Zn, and the highest positive loading for As; Hg has no influence.

In the first PCA (Fig. 3A), there was a tendency to cluster the specifications by species, since the overlap between hedgehogs and badgers (circled) is the smallest of all. The Kruskal-Wallis test results or the species factor were consistent with these findings, since statistically significant differences were observed for PTEs (*p*<0.05). Therefore, the differences may be due to the fact that they are two completely different species, and in each species the pattern of accumulation of PTEs will be different. Badgers tend to follow the pattern established by Zn and Pb, while hedgehogs are attributed to Cd and Hg. The hypotheses we put forward are that these trends may be generally influenced by dietary habits. Behaviour could be another parameter, as hedgehogs are insectivorous linked to (sub)urban areas being affected to a greater extend by exposure to Cd and Hg (D'Havé et al. 2006). Another parameter is the physiological function, for example hedgehogs are hibernators, and during this period, the stress caused by PTEs body burden may be crucial (Rautio et al. 2010). Finally, it is interesting to note the behaviour of hepatic Zn, which increases when it decreases in the kidney and vice versa, in spite this did not result in a significant negative correlation. Badgers tended to follow this trend possibly because omnivores accumulate Zn first in the liver followed by the kidney, and also because one of the places where Zn homeostasis is maintained is in the hepatic tissue (Kosik-Bogacka and Łanocha-Arendarczyk 2019).

### Age and sex influence

**Fig. 4:**
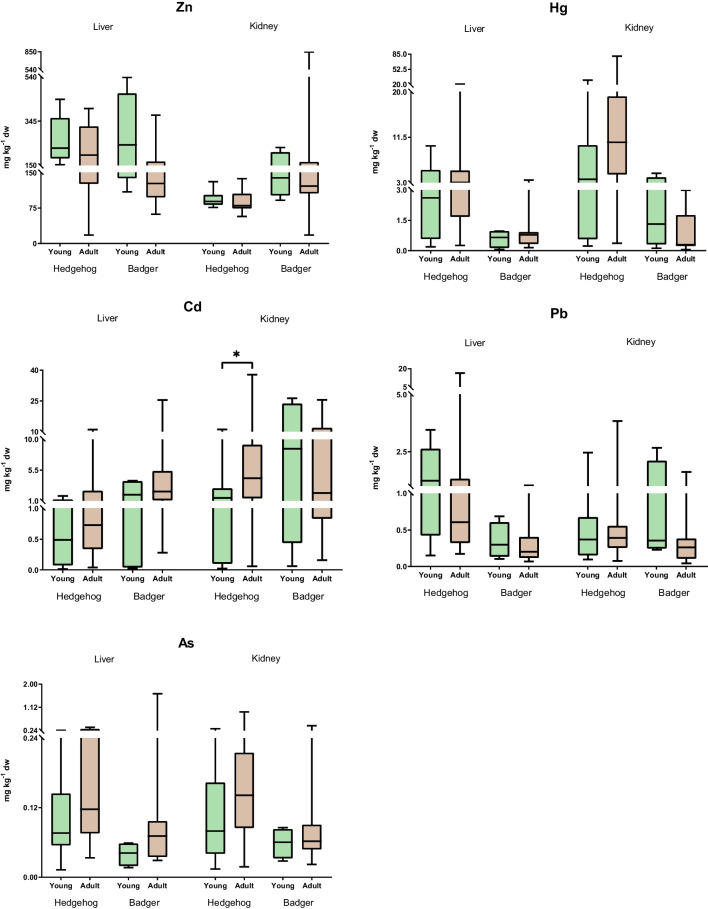
Distribution of Zn, Hg, Cd, Pb and As (mg kg^-1^ dw) in the liver and kidneys for both species, European hedgehog and Eurasian badger, according to age. Box plots represent median values and 25–75 percentile. Significance levels were *p<0.05

The age of an organism often influences the accumulation of PTEs like Cd. In our study, consistent with expectations, adult hedgehogs showed more significant Cd accumulation in their kidneys compared to their younger counterparts, with mean concentrations being 6.99 and 2.45 mg kg^-1^ dw; respectively (*p*<0.05) (Fig. 4). This age-related accumulation can be attributed to the prolonged exposure coupled with metals’ extended biological half-life (Cooke 2011). Another contributing factor is the detoxifying nature of nephritic tissue. Specifically, to neutralize Cd’s toxic effects, the body Cd-MT (metallothioneins) complexes (Sánchez-Chardi et al. 2007). While our results demonstrate this pattern in hedgehogs, similar tendencies, albeit not statistically significant, were also observed in badgers, particularly concerning As accumulation. Previous studies corroborate our findings, having reported hepatic and renal Cd accumulations in adult hedgehogs. Similar patterns were also observed for other metals like Hg, Pb, and As (Rautio et al. 2010; Dahmardeh Behrooz et al. 2022; Jota Baptista et al. 2023). However, while the PTE concentrations detected in this study are relatively low, understanding their significance would require insight into the concentrations in the subsequent trophic level. In the absence of such data, drawing a conclusive level of concern remains challenging. Nonetheless, our findings highlight potential exposure risks to apex predators that primarily feed on older animals, hinting at bioaccumulation effects up the food chain.

Contrary trends were observed in the levels of Zn in hedgehogs and, generally, in badgers where the young tended to accumulate relatively more PTEs than adults. It has been suggested that young animals are more susceptible to Zn accumulation than older animals due to their high uptake of food for energy requirements and their high absorption rate (Kosik-Bogacka and Łanocha-Arendarczyk 2019). Similar findings have been reported for hedgehogs from Finland, in which the Zn concentration decreased with age (Rautio et al. 2010). However, in badgers, Van Den Brink and Ma (1998) found an age-influence in the oldest from the Netherlands, which had accumulated significantly high levels of Cd, Zn and Pb. Renal Pb accumulation in adult badgers has also been recognised (Ma 2011), although this was not observed in our study. The fact that young badgers have accumulated more Pb than adults may be due to the fact that Pb is an analogue of calcium which is rapidly exchanged, being higher in juveniles when compared to adults (Skerfving and Bergdahl 2015). When the age factor was evaluated, PCA showed an overlap between young and adults (Fig. 3B). This plot determines that the factor does not have an influence and is therefore not acting on the set of variables to. However, it seems that young animals tended to show a marked trend for Hg, Cd and As.

Data about gender-related metal accumulation in European hedgehog and Eurasian badger are scarce. Even though gender has a physiological function in metal accumulation, as abovementioned in the introduction section, little is known about its effects as a consequence of differences in hormonal and reproductive state, gene expression and metabolic rate between males and females (Burger 2007). In the present study, we did not observe statistically significant differences in either species (*p*>0.05) (Supplementary materials; Fig. 1S). In addition, PCA do not shows a significant trend on PTEs concentrations when the sex factor was considered, being data almost overlap (Supplementary materials; Fig. 2S). Thus, inter-population variation caused by differences in exposure and uptake of elements may also have influenced this discordant result. Generally, as reflected in the figures, we observed a slight trend of PTEs accumulation in females compared to males. Moreover, females tended to show a marked trend for Hg and Cd, while Pb and Zn were for males. Some authors claim that these differences are attributed to reduced dispersal rate when rearing pups, leaving them closer to areas contaminated by point sources or differences between feeding habits (Dibbern et al. 2021; Sánchez et al. 2022). These trends are consistent with a recent study developed by Jota Baptista et al. (2023), where significant differences in Pb concentration in the liver of hedgehogs from Portugal were observed; however, the opposite of those showed by Dahmardeh Behrooz et al. (2022) in Brant hedgehogs, whose Hg levels in the kidney were higher in males when compared to females; in another study, no difference between males and females was observed (Rautio et al. 2010). Nonetheless, the results obtained in the present study are in accordance with those carried out in insectivorous small mammals such as European mole, and even in mammal that are higher up the trophic chain (Komarnicki 2000; Scheirs et al. 2006; López-Alonso et al. 2007; Millán et al. 2008).

In the present study, male badgers showed a slight tendency to accumulate As and Cd, while females accumulated Zn and Pb, but this was not statistically different (*p*>0.05). Low levels in females may be attributed to the transfer of PTEs to the foetus during gestation and lactation (Shore et al. 2011). In contrast to these results, Cd accumulation in female badgers has been reported (Van Den Brink and Ma 1998; Millán et al. 2008). Thus, there is no clear evidence about the relationship between gender and metal accumulation, suggesting that the accumulation can differ among species, seasons or reproductive status. However, these results in badgers are not consistent with that of other mesocarnivores, such as the American mink (*Neovison vison*) (Mayack 2012). Sex-related differences in metal accumulation have also been identified in other biomonitoring investigations, with females exhibiting higher metal concentrations compared to males in terrestrial wild mammals (Hernández-Moreno et al. 2013). Some researchers have attributed this phenomenon to differences in the efficiency of metallothionein (MT) synthesis in females (Cooke 2011). While the influence of sex on metal accumulation has been explored to a limited extent, it underscores the need to elucidate the toxicokinetics of individual inorganic elements in males and females across different species. This consideration should be incorporated into future biomonitoring studies (Neila et al. 2017).

## Conclusions

Our research provides current levels of potentially toxic elements (PTEs) in wildlife terrestrial ecosystems in NW Spain. This is the first contribution to report baseline Hg levels in the European hedgehog in the Iberian Peninsula, finding these concentrations above the threshold value for serious health effects, and nephritic reference levels in terrestrial wild mammals in our study area were also determined for the first time. This research also showed an age-related in Cd accumulation and this physiological factor should, therefore, be considered in the assessment of PTEs body burdens. However, sex had no effect, so it is necessary to improve our knowledge on this factor. In addition, our results should be included in a database with baseline values for PTEs bioaccumulation in European insectivores and mesocarnivores. The data obtained in this ecotoxicological study have significant potential applications in wildlife management, environmental quality improvement and environmental risk assessment in the northwest of the Iberian Peninsula. Moreover, our results underline the importance of proper environmental management and the need for further research to determine the exact sources of this contamination. In summary, this study highlights the importance of addressing and mitigating PTEs contamination in terrestrial ecosystems in NW Spain. This knowledge is fundamental for the conservation of biodiversity and the protection of environmental quality in this region. It is therefore necessary to monitor and understand the presence of PTEs in local ecosystems, and to stress the need for appropriate conservation and environmental management measures to protect both wildlife and the natural environment.

### Supplementary information

ESM 1 Fig. 1S. Distribution of Zn, Hg, Cd, Pb and As (ppm dw) in the liver and kidneys for both species, European hedgehog and Eurasian badger, according to sex. Box plots represent median values and 25–75 percentile. Significance levels were *p<0.05. (PDF 12 kb)

ESM 2 Fig. 3S. Biplot of PC1 versus PC2 with loadings of Zn, Hg, Cd, Pb and As measured in liver and kidney of 39 samples of European hedgehog and 20 samples of European Badger. The colours represent the sex (red: female, blue: male). The total variance explained by the first two principal axes is 55%. (PDF 6 kb)

## Data Availability

All data and materials will be made available upon request.

## References

[CR1] Adrian WJ, Stevens ML (1979). Wet versus dry weights for heavy metal toxicity determinations in duck liver. J Wildl Dis.

[CR2] Al Sayegh Petkovšek S, Kopušar N, Kryštufek B (2014). Small mammals as biomonitors of metal pollution: a case study in Slovenia. Environ Monit Assess.

[CR3] Ali H (2018). Khan E (2018) What are heavy metals? Long-standing controversy over the scientific use of the term ‘heavy metals’ – proposal of a comprehensive definition. Toxicol Environ Chem.

[CR4] Ali H, Khan E (2019). Trophic transfer, bioaccumulation, and biomagnification of non-essential hazardous heavy metals and metalloids in food chains/webs-Concepts and implications for wildlife and human health. Hum Ecol Risk Assess.

[CR5] Alleva E, Francia N, Pandolfi M, De Marinis AM, Chiarotti F, Santucci D (2006). Organochlorine and heavy-metal contaminants in wild mammals and birds of Urbino-Pesaro Province, Italy: an analytic overview for potential bioindicators. Arch Environ Contam Toxicol.

[CR6] ATSDR (2022). Substance priority list.

[CR7] Baranowska-Bosiacka I, Korbecki J, Marchlewicz M, Kalisińska E (2019). Lead, Ld. Mammals and Birds as Bioindicators of Trace Element Contaminations in Terrestrial Environments.

[CR8] Beernaert J, Scheirs J, Leirs H, Blust R, Verhagen R (2007). Non-destructive pollution exposure assessment by means of wood mice hair. Environ. Pollut (Barking Essex : 1987).

[CR9] Beyer WN, Codling EE, Rutzke MA (2018). Anomalous bioaccumulation of lead in the earthworm *Eisenoides lonnbergi* (Michaelsen). Environ Toxicol Chem.

[CR10] Bilandžić N, Dežđek D, Sedak M, Dokić M, Simić B, Rudan N, Brstilo M, Lisicin T (2012). Trace elements in tissues of wild carnivores and omnivores in Croatia. Bull Environ Contam Toxicol.

[CR11] Binkowski LJ, Kalisińska E (2019). Arsenic, As. Mammals and Birds as Bioindicators of Trace Element. Contaminations in Terrestrial Environments.

[CR12] Boening DW (2000). Ecological effects, transport, and fate of mercury: a general review. Chemosphere.

[CR13] Brand AF, Hynes J, Walker LA, Glόria Pereira M, Lawlor AJ, Williams RJ, Shore RF, Chadwick EA (2020). Biological and anthropogenic predictors of metal concentration in the Eurasian otter, a sentinel of freshwater ecosystems. Environ Pollut (Barking, Essex: 1987).

[CR14] Bukovjan K, Toman A, Kutlvašr K, Marada P, Kodet R, Sláma P, Křikava L (2014). Contents of chemical elements in tissues of European badger (*Meles meles*) affected by ovarian tumour - a case report. Acta Vet Brno.

[CR15] Burger J (2007). A framework and methods for incorporating gender-related issues in wildlife risk assessment: gender-related differences in metal levels and other contaminants as a case study. Environ Res.

[CR16] Charkiewicz AE, Backstrand JR (2020). Lead toxicity and pollution in Poland. Int J Environ Res Public Health.

[CR17] Chormare R, Kumar MA (2022). Environmental health and risk assessment metrics with special mention to biotransfer, bioaccumulation and biomagnification of environmental pollutants. Chemosphere.

[CR18] Cooke JA, Beyer WN, Meador JP (2011). Cadmium in Small Mammals. Environmental Contaminants in Biota. Interpreting Tissue Concentrations.

[CR19] Dahmardeh Behrooz R, Poma G, Barghi M (2022). Non-destructive mercury exposure assessment in the Brandt's hedgehog (*Paraechinus hypomelas*): spines as indicators of endogenous concentrations. Environ Sci Pollut Res.

[CR20] De La Peña-Lastra S, Pérez-Alberti A, Otero XL (2019). Enrichment of trace elements in colonies of the yellow-legged gull (*Larus michahellis*) in the Atlantic Islands National Park (Galicia-NW Spain). Sci Total Environ.

[CR21] D'Havé H, Scheirs J, Mubiana VK, Verhagen R, Blust R, De Coen W (2005). Nondestructive pollution exposure assessment in the European hedgehog (*Erinaceus europaeus*): I. Relationships between concentrations of metals and arsenic in hair, spines, and soil. Environ Toxicol Chem.

[CR22] D'Havé H, Scheirs J, Mubiana VK, Verhagen R, Blust R, De Coen W (2006). Non-destructive pollution exposure assessment in the European hedgehog (*Erinaceus europaeus*): II. Hair and spines as indicators of endogenous metal and As concentrations. Environ Pollut, (BarkingEssex: 1987).

[CR23] Dibbern M, Elmeros M, Dietz R, Søndergaard J, Michelsen A, Sonne C (2021). Mercury exposure and risk assessment for Eurasian otters (*Lutra lutra*) in Denmark. Chemosphere.

[CR24] Eisler R (1993). Zinc hazards to fish, wildlife, and invertebrates: a synoptic review.

[CR25] Eisler R (2004). Mercury hazards from gold mining to humans, plants, and animals. Rev Environ Contam Toxicol.

[CR26] ETS 104 (1979). Conservation of wildlife and natural habitats, Bern 19.IX.1979.

[CR27] Fritsch C, Cosson RP, Coeurdassier M, Raoul F, Giraudoux P, Crini N, de Vaufleury A, Scheifler R (2010). Responses of wild small mammals to a pollution gradient: host factors influence metal and metallothionein levels. Environ Pollut (Barking, Essex: 1987).

[CR28] Gall JE, Boyd RS, Rajakaruna N (2015). Transfer of heavy metals through terrestrial food webs: a review. Environ Monit Assess.

[CR29] Giráldez P, Crujeiras RM, Fernández JÁ, Aboal JR (2022). Establishment of background pollution levels and spatial analysis of moss data on a regional scale. Sci Total Environ.

[CR30] González XI, Aboal JR, Fernández JA, Carballeira A (2008). Evaluation of some sources of variability in using small mammals as pollution biomonitors. Chemosphere.

[CR31] Goretti E, Pallottini M, Cenci Goga BT, Selvaggi R, Petroselli C, Vercillo F, Cappelletti D (2018). Mustelids as bioindicators of the environmental contamination by heavy metals. Ecol.

[CR32] Heimstad ES, Nygård T, Herzke D, Bohlin-Nizzetto P (2019). Environmental pollutants in the terrestrial and urban environment 2018.

[CR33] Hernández LM, González MJ, Rico MC, Fernández MA, Baluja G (1985). Presence and biomagnification of organochlorine pollutants and heavy metals in mammals of Doñana National Park (Spain), 1982-1983. J Environ Sci Health Part B.

[CR34] Hernández-Moreno D, de la Casa RI, Fidalgo LE, Llaneza L, Soler Rodríguez F, Pérez-López M, López-Beceiro A (2013). Non-invasive heavy metal pollution assessment by means of Iberian wolf (*Canis lupus signatus*) hair from Galicia (NW Spain): a comparison with invasive samples. Environ Monit Assess.

[CR35] Jota Baptista C, Seixas F, Gonzalo-Orden JM, Oliveira PA (2022). Biomonitoring metals and metalloids in wild mammals: invasive versus non-invasive sampling. Environ Sci Pollut Res.

[CR36] Jota Baptista C, Seixas F, Gonzalo-Orden JM, Oliveira PA (2022). Biomonitoring of heavy metals and metalloids with wild mammals in the Iberian Peninsula: a systematic review. Environ Rev.

[CR37] Jota Baptista C, Seixas F, Gonzalo-Orden JM, Patinha C, Pato P, Ferreira da Silva E, Casero M, Brazio E, Brandão R, Costa D, Mateus TL, Oliveira PA (2023). High Levels of Heavy Metal(loid)s Related to Biliary Hyperplasia in Hedgehogs (*Erinaceus europaeus*). Animals.

[CR38] Jota Baptista CV, Seixas F, Gonzalo-Orden JM, Oliveira PA (2021). Can the European hedgehog (*Erinaceus europaeus*) be a sentinel for One Health concerns?. Biologics.

[CR39] Kalisińska E, Kalisińska E (2019). Endothermic animals as biomonitors of terrestrial environments. Mammals and birds as bioindicators of trace element contaminations in terrestrial environments, an Ecotoxicological Assessment of the Northern Hemisphere.

[CR40] Kalisińska E, Kot K, Łanocha-Arendarczyk N (2023). Red fox as a potential bioindicator of metal contamination in a European environment. Chemosphere.

[CR41] Kalisinska E, Lanocha-Arendarczyk N, Podlasinska J (2021) Current and historical nephric and hepatic mercury concentrations in terrestrial mammals in Poland and other European countries. Sci Total Environ 775:145808. 10.1016/j.scitotenv.2021.145808 10.1016/j.scitotenv.2021.145808 33621879

[CR42] Komarnicki GJ (2000). Tissue, sex and age specific accumulation of heavy metals (Zn, Cu, Pb, Cd) by populations of the mole (*Talpa europaea L.*) in a central urban area. Chemosphere.

[CR43] Kosik-Bogacka D, Łanocha-Arendarczyk N, Kalisińska E (2019). Zinc, Zn. Mammals and Birds as Bioindicators of Trace Element Contaminations in Terrestrial Environments an Ecotoxicological Assessment of the Northern Hemisphere.

[CR44] Lanszki J, Orosz E, Sugár L (2009). Metal levels in tissues of Eurasian otters (*Lutra lutra*) from Hungary: variation with sex, age, condition and location. Chemosphere.

[CR45] Lê S, Josse J, Husson F (2008). FactoMineR: A Package for Multivariate Analysis. J Stat Softw.

[CR46] López-Alonso M, Miranda M, García-Partida P, Cantero F, Hernández J, Benedito JL (2007). Use of dogs as indicators of metal exposure in rural and urban habitats in NW Spain. Sci Total Environ.

[CR47] Ma WC, Beyer WN, Heinz GH, Redmon-Norwood AW (1996). Lead in mammals. Environmental Contaminants in Wildlife.

[CR48] Ma WC, Beyer WN, Meador JP (2011). Lead in mammals. Environmental Contaminants in Biota. Interpreting Tissue Concentrations.

[CR49] Marques CC, Sánchez-Chardi A, Gabriel SI, Nadal J, Viegas-Crespo AM, da Luz MM (2007). How does the greater white-toothed shrew, *Crocidura russula*, responds to long-term heavy metal contamination? -- A case study. Sci Total Environ.

[CR50] Mayack DT (2012). Hepatic mercury, cadmium, and lead in mink and otter from New York State: monitoring environmental contamination. Environ Monit Assess.

[CR51] Millán J, Mateo R, Taggart MA, López-Bao JV, Viota M, Monsalve L, Camarero PR, Blázquez E, Jiménez B (2008). Levels of heavy metals and metalloids in critically endangered Iberian lynx and other wild carnivores from Southern Spain. Sci Total Environ.

[CR52] Mullineaux ST, Redpath S, Ogle N, McKinley JM, Marks NJ, Scantlebury DM, Doherty R (2021). Potentially toxic element accumulation in badgers (*Meles meles*): a compositional approach. Sci Total Environ.

[CR53] Nardiello V, Fidalgo LE, López-Beceiro A, Bertero A, Martínez-Morcillo S, Míguez-Santiyán MP, Soler Rodríguez F, Caloni F, Pérez-López M (2019). Metal content in the liver, kidney, and feathers of Northern gannets, Morus bassanus, sampled on the Spanish coast. Environ Sci Pollut Res Int.

[CR54] Neila C, Hernández-Moreno D, Fidalgo LE, López-Beceiro A, Soler Rodríguez F, Pérez-López M (2017). Does gender influence the levels of heavy metals in liver of wild boar?. Ecotoxicol Environ Saf.

[CR55] NRC (2005). Mineral Tolerance of Animals.

[CR56] Osuna-Martínez CC, Armienta MA, Bergés-Tiznado ME, Páez-Osuna F (2021). Arsenic in waters, soils, sediments, and biota from Mexico: An environmental review. Sci Total Environ.

[CR57] Ozimec S, Florijančić T, Milin Radić S, Bilandžić N, Bošković I (2015). Bioaccumulation of cadmium and lead in the European badger (*Meles meles* L.) from the Croatian Danube region. J Environ Prot Ecol.

[CR58] Ozimec S, Milin Radić S, Florijančić T, Bošković I, Gross-Bošković A (2017). Mercury level in tissues of European badger (*Meles meles* L.) from Eastern Slavonia. Croatia Int J Food Biosyst Eng.

[CR59] Peakall D, Burger J (2003). Methodologies for assessing exposure to metals: speciation, bioavailability of metals, and ecological host factors. Ecotoxicol Environ Saf.

[CR60] Pereira R, Pereira ML, Ribeiro R, Gonçalves F (2006). Tissues and hair residues and histopathology in wild rats (*Rattus rattus* L.) and Algerian mice (*Mus spretus* Lataste) from an abandoned mine area (Southeast Portugal). Environ Poll (Barking, Essex: 1987).

[CR61] Pérez-López M, Soler Rodríguez F, Hernández-Moreno D, Rigueira L, Fidalgo LE, López-Beceiro A (2016). Bioaccumulation of cadmium, lead and zinc in liver and kidney of red fox (*Vulpes vulpes*) from NW Spain: influence of gender and age. Toxicol Environ Chem.

[CR62] Peterson SH, Ackerman JT, Hartman CA, Casazza ML, Feldheim CL, Herzog MP (2020). Mercury exposure in mammalian mesopredators inhabiting a brackish marsh. Environ Pollut (Barking, Essex : 1987).

[CR63] Petrović Z, Teodorović V, Djurić S, Milićević D, Vranić D, Lukić M (2014). Cadmium and mercury accumulation in European hare (*Lepus europaeus*): age-dependent relationships in renal and hepatic tissue. Environ Sci Pollut Res Int.

[CR64] R Core Team (2023). R: A language and environment for statistical computing.

[CR65] Raj D, Maiti SK (2020). Sources, bioaccumulation, health risks and remediation of potentially toxic metal(loid)s (As, Cd, Cr, Pb and Hg): an epitomised review. Environ Monit Assess.

[CR66] Rautio A, Kunnasranta M, Valtonen A, Ikonen M, Hyvärinen H, Holopainen IJ, Kukkonen JV (2010). Sex, age, and tissue specific accumulation of eight metals, arsenic, and selenium in the European hedgehog (*Erinaceus europaeus*). Arch Environ Contam Toxicol.

[CR67] Sánchez CA, Penrose MT, Kessler MK, Becker DJ, McKeown A, Hannappel M, Boyd V, Camus MS, Padgett-Stewart T, Hunt BE, Graves AF, Peel AJ, Westcott DA, Rainwater TR, Chumchal MM, Cobb GP, Altizer S, Plowright RK, Boardman WSJ (2022). Land use, season, and parasitism predict metal concentrations in Australian flying fox fur. Sci Total Environ.

[CR68] Sánchez-Chardi A, Marques CC, Nadal J, da Luz MM (2007). Metal bioaccumulation in the greater white-toothed shrew, *Crocidura russula*, inhabiting an abandoned pyrite mine site. Chemosphere.

[CR69] Sánchez-Chardi A, Nadal J (2007). Bioaccumulation of metals and effects of landfill pollution in small mammals. Part I. The greater white-toothed shrew, *Crocidura russula*. Chemosphere.

[CR70] Scheirs J, De Coen A, Covaci A, Beernaert J, Kayawe VM, Caturla M, De Wolf H, Baert P, Van Oostveldt P, Verhagen R, Blust R, De Coen W (2006). Genotoxicity in wood mice (*Apodemus sylvaticus*) along a pollution gradient: exposure-, age-, and gender-related effects. Environ Toxicol Chem.

[CR71] Shore RF (1995). Predicting cadmium, lead and fluoride levels in small mammals from soil residues and by species-species extrapolation. Environ Pollut (Barking, Essex: 1987).

[CR72] Shore RF, Douben PET (1994). Predicting ecotoxicological impacts of environmental contaminants on terrestrial small mammals. Rev Environ Contam Toxicol.

[CR73] Shore RF, Pereira MG, Walker LA, Thompson DR, Beyer WN, Meador JP (2011). Mercury in nonmarine birds and mammals. Environmental Contaminants in Biota. Interpreting Tissue Concentrations.

[CR74] Skerfving S, Bergdahl IA, Nordberg GF, Fowler BA, Nordberg M (2015). Lead. Handbook on the Toxicology of Metals.

[CR75] Talmage SS, Walton BT (1991). Small mammals as monitors of environmental contaminants. Rev Environ Contam Toxicol.

[CR76] Tomza-Marciniak A, Pilarczyk B, Marciniak A, Udała J, Bąkowska M, Pilarczyk R, Kalisińska E (2019). Cadmium, Cd. Mammals and Birds as Bioindicators of Trace Element Contaminations in Terrestrial Environments.

[CR77] Van Den Brink NW, Ma WC (1998). Spatial and temporal trends in levels of trace metals and PCBs in the European badger *Meles meles* (L., 1758) in The Netherlands: implications for reproduction. Sci Total Environ.

[CR78] Vermeulen F, D'Havé H, Mubiana VK, Van den Brink NW, Blust R, Bervoets L, De Coen W (2009). Relevance of hair and spines of the European hedgehog (*Erinaceus europaeus*) as biomonitoring tissues for arsenic and metals in relation to blood. Sci Total Environ.

[CR79] Walker CH, Sibly RM, Hopkin SP, Peakall DB (2012). Principles of Ecotoxicology.

[CR80] Wei T, Simko V (2021). R package 'corrplot': Visualization of a Correlation Matrix. (Version 0.92).

[CR81] Wickham H (2016). ggplot2: Elegant Graphics for Data Analysis.

[CR82] Wobeser G, Nielsen NO, Schiefer B (1976). Mercury and Mink. II. Experimental methyl mercury intoxication. Can J Comp Med.

[CR83] Yan X, Wang J, Zhu L, Wang J, Li S, Kim YM (2021). Oxidative stress, growth inhibition, and DNA damage in earthworms induced by the combined pollution of typical neonicotinoid insecticides and heavy metals. Sci Total Environ.

[CR84] Zarrintab M, Mirzaei R (2017). Evaluation of some factors influencing on variability in bioaccumulation of heavy metals in rodents species: *Rombomys opimus* and *Rattus norvegicus* from central Iran. Chemosphere.

[CR85] Zhang G, Yin D, He T, Xu Y, Ran S, Zhou X, Tian X, Wang Y (2021). Mercury bioaccumulation in freshwater snails as influenced by soil composition. Bull Environ Contam Toxicol.

