# Correction to: Metal and metalloid concentrations in wild mammals from SW Europe: European hedgehog (*Erinaceus europaeus*) and badger (*Meles meles*)

**DOI:** 10.1007/s11356-023-31077-4

**Published:** 2023-11-18

**Authors:** Javier García‑Muñoz, Nunzio Antonio Cacciola, Federico Plazzi, María Prado Míguez‑Santiyán, Francisco Soler Rodríguez, Ana López‑Beceiro, Luis Eusebio Fidalgo, Salomé Martínez‑Morcillo, Marcos Pérez‑López

**Affiliations:** 1 Toxicology Area, Faculty of Veterinary Medicine, (Universidad de Extremadura), 10003 Cáceres, Spain; 2 Department of Veterinary Medicine and Animal Production, University of Naples Federico II, Via F. Delpino 1, 80137 Naples, Italy; 3 Department of Biologia Evoluzionistica Sperimentale, University of Bologna, Via Selmi 3, 40126 Bologna, Italy; 4 Department of Veterinary Clinical Sciences, Faculty of Veterinary Medicine (USC), 27003 Lugo, Spain

**Correction to: Environmental Science and Pollution Research**

10.1007/s11356-023-30615-4

Missing Acknowledgment text in the published proof.

The Original article has been corrected.

