# Supplementary figures and images for: Metal and metalloid concentrations in wild mammals from SW Europe: European hedgehog (Erinaceus europaeus) and badger (Meles meles)
Source: Environ Sci Pollut Res Int. 2023 Nov 3;30(56):118855–70. doi: 10.1007/s11356-023-30615-4 (PMC10697895; doi:10.1007/s11356-023-30615-4)

## Zn

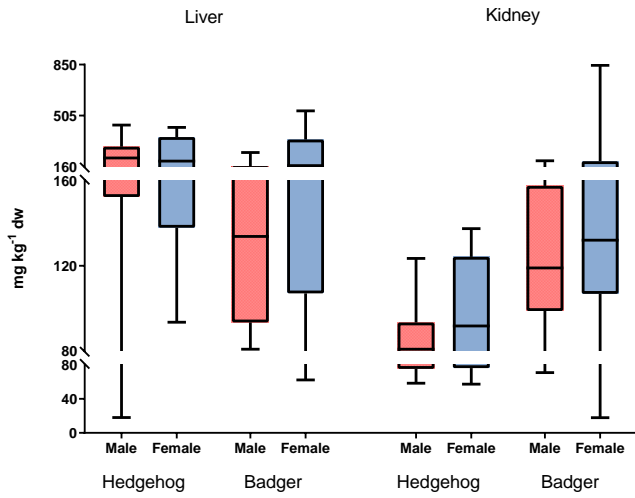

## Hg

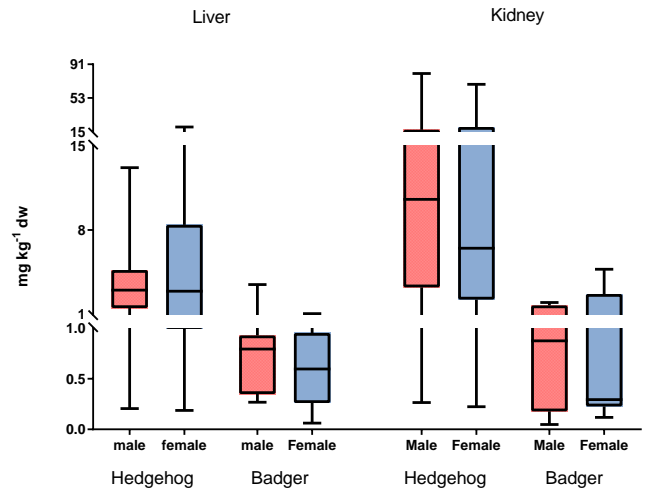

## Cd

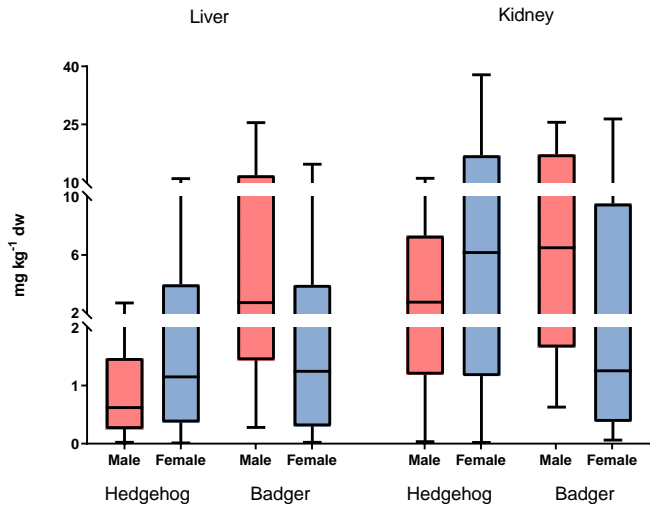

## Pb

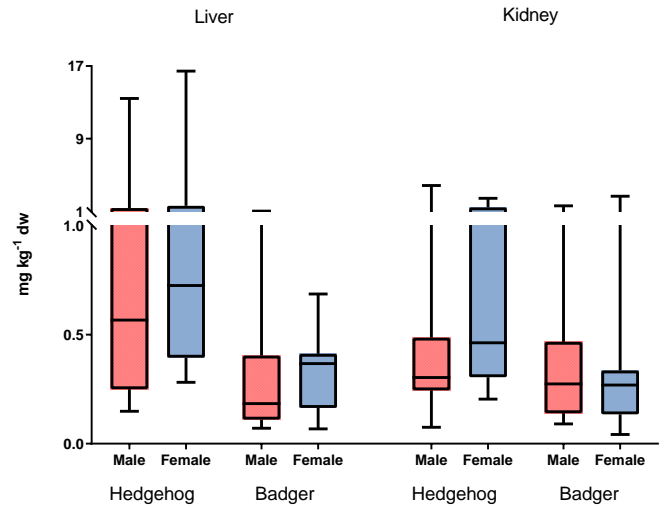

## As

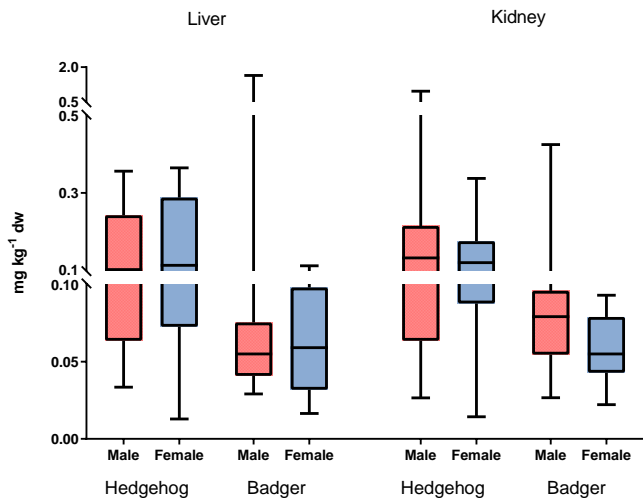

Supplement: Supplementary file 1 — Fig. 1S. Distribution of Zn, Hg, Cd, Pb and As (ppm dw) in the liver and kidneys for both species, European hedgehog and Eurasian badger, according to sex. Box plots represent median values and 25–75 percentile. Significance levels were *p<0.05. (PDF 12 kb) [file 11356_2023_30615_MOESM1_ESM.pdf]

Metals (N=59)

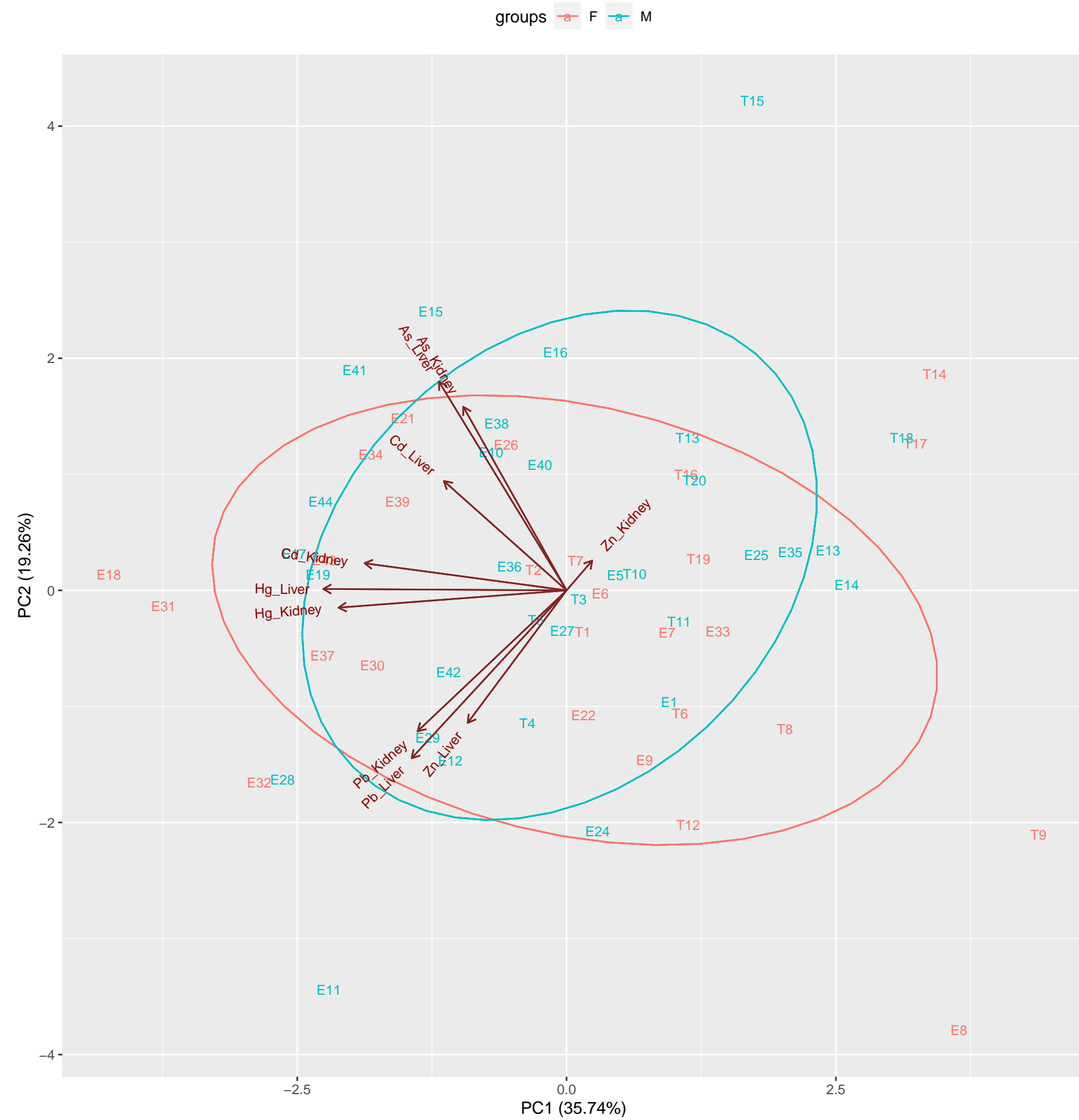

Supplement: Supplementary file 2 — Fig. 3S. Biplot of PC1 versus PC2 with loadings of Zn, Hg, Cd, Pb and As measured in liver and kidney of 39 samples of European hedgehog and 20 samples of European Badger. The colours represent the sex (red: female, blue: male). The total variance explained by the first two principal axes is 55%. (PDF 6 kb) [file 11356_2023_30615_MOESM2_ESM.pdf]
